# Mesoscopic Interference of Rotated Spins in Graphene Coupled to High-Spin–Orbit-Coupling Substrates

**DOI:** 10.1021/acsami.5c15694

**Published:** 2025-10-20

**Authors:** Kazushi Yokoi, Ratchanok Somphonsane, Harihara Ramamoorthy, Nargess Arabchigavkani, Keke He, Bilal Barut, Shenchu Yin, Michael D. Randle, Ripudaman Dixit, Jubin Nathawat, Jonas Fransson, Gil-Ho Kim, Kenji Watanabe, Takashi Taniguchi, Jonathan P. Bird, Nobuyuki Aoki

**Affiliations:** † Department of Materials Science, 12737 Chiba University, Inage-ku, Chiba 263-8522, Japan; ‡ Department of Physics, School of Science, 61794 King Mongkut’s Institute of Technology Ladkrabang, Bangkok 10520, Thailand; § Department of Electronics Engineering, School of Engineering, King Mongkut’s Institute of Technology Ladkrabang, Bangkok 10520, Thailand; ∥ Department of Electrical Engineering, University at Buffalo, The State University of New York, Buffalo, New York 14260, United States; ⊥ Department of Physics and Astronomy, 8097 Uppsala University, Box 534, Uppsala SE-751 21, Sweden; # Department of Electrical and Computer Engineering & Sungkyunkwan Advanced Institute of Nanotechnology (SAINT), Sungkyunkwan University, Suwon 16419, Republic of Korea; ¶ Research Center for Functional Materials, 52747 National Institute for Materials Science, Tsukuba 305-0044, Japan; ∇ International Center for Material Nano-Architectonics, National Institute for Materials Science, Tsukuba 305-0044, Japan

**Keywords:** 2D semiconductors, spin−orbit coupling, weak antilocalization, mesoscopics, graphene, transition-metal dichalcogenides

## Abstract

We explore the manifestations of spin rotation in graphene in proximity with two different types of high-spin–orbit-coupling (SOC) materials (ferromagnetic Co and nominally diamagnetic WSe_2_). Using weak antilocalization (WAL) as a probe of the induced rotation, we demonstrate that spin interference exhibits a highly stochastic (nonself-averaging) character in the mesoscopic limit. At low temperatures (<20 K), the spin rotation is manifested as a zero-bias peak (or zero-bias anomaly, ZBA) in the differential conductance, a feature that, as expected for WAL, is suppressed by fairly modest magnetic fields (<∼10^2^ mT). The ZBA moreover exhibits a stochastic variation when a gate voltage is used to sweep the Fermi level through the graphene bands, with ranges for which the antilocalization is either prominent or strongly suppressed. This mesoscopic character is exhibited by both of the studied systems, whose ZBA is also damped in similar fashion with increasing temperature. We thus provide fundamental insight into the nonensemble-averaged (nonself-averaged) character of spin interference in mesoscopic systems with strong SOC and, more specifically, into how the details of spin rotation are impacted by external gating. This understanding may ultimately enable the efficient modulation of spin currents in future spintronic devices.

## Graphical abstract

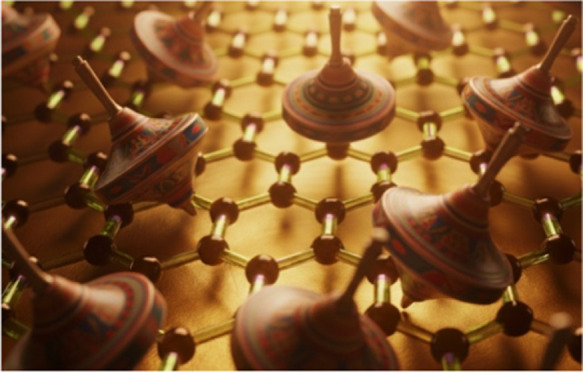

## 1 Introduction

Spin–orbit coupling (SOC) in a semiconductor is responsible for a deterministic rotation of the spin of the electron as it propagates through a crystal. As such, it offers a pathway to the controlled manipulation of spin in various classical^1^ and quantum^2^ devices. The possibilities for exploiting SOC in such applications have been greatly expanded in recent years with the advent of two-dimensional (2D) semiconductors. These atomically thin materials can be integrated^3–15^ with functional substrates (including ferromagnets,^3,4,6,14^ multiferroics,^7^ complex oxides,^15^ and topological insulators^5^), to generate extrinsic SOC via proximity coupling. This concept of engineering SOC via heterogeneous material integration offers the potential to significantly advance the development of spintronics.^16^

A powerful tool for detecting spin rotation induced by spin–orbit scattering is provided by weak antilocalization (WAL), a quantum-transport effect that arises from the interference of pairs of rotated spins that propagate in opposite directions around the same closed scattering paths.^17,18^ In thin metallic films with strong SOC, ensemble averaging of the spin interference over a broad distribution of such paths results in a relative phase rotation between the counterpropagating spins that is most typically close to 2π (rather than zero).^17^ Since spin-1/2 particles have a rotational periodicity of 4π,^19^ this antilocalization reduces the probability of backscattering and thus leads to an enhancement of the conductance (at zero magnetic field).^18^

While a long history of studies of WAL has established the details of this ensemble-averaged phenomenon in bulk-like materials with strong SOC, the nature of spin rotation in the mesoscopic limit is less well understood. In this regime, quantum transport exhibits a coherent character on a scale comparable to the system size, and ensemble averaging is no longer guaranteed.^20^ To address the nature of spin rotation in such systems, in this work, we explore its manifestations in graphene that we couple to two different types of high-SOC material (ferromagnetic Co and nominally diamagnetic WSe_2_). The use of graphene in these studies is advantageous for two key reasons. First, the carrier concentration in graphene can be externally gated, allowing for investigations of spin rotation as the Fermi level is swept through its Dirac bands. Second, in its native state, graphene exhibits only weak SOC and so any observed signatures of this effect must therefore arise through external means (i.e., through proximity). In the experiments that we perform here, we provide evidence of a highly stochastic (nonself-averaging) character to spin rotation in the mesoscopic limit. At low temperatures (<20 K), the spin rotation is manifested as a zero-bias peak (or zero-bias anomaly, ZBA) in the differential conductance, a feature that, as expected for WAL,^17,18^ is suppressed by fairly modest magnetic fields (<∼10^2^ mT). The ZBA moreover exhibits a stochastic variation when a gate voltage (*V*_g_) is used to sweep the Fermi level through the graphene bands, with ranges for which the antilocalization is either prominent or strongly suppressed. This mesoscopic character is exhibited by both studied systems, whose ZBA is also damped in similar fashion with increasing temperature. Our demonstrations thus provide fundamental insight into the nonensemble-averaged character of spin interference in mesoscopic systems with strong SOC and, more specifically, into how the details of spin rotation may be externally gated. This understanding may ultimately enable the efficient modulation of spin currents in future spintronic devices.^1,2^

## 2 Results

### 2.1 The Experimental Systems

In this work, we explore the signatures of mesoscopic spin rotation due to extrinsic SOC in two very different systems. The first of these is graphene in proximity to a ferromagnet [Co, see Figure 1a], a combination that we have studied previously^14^ but for which we present new experimental results here. The second is a van der Waals heterostructure in which graphene is stacked upon monolayer WSe_2_ [Figure 2a,b], the latter of which is characterized by strong SOC.^21–31^ Through a detailed comparison of the behavior exhibited by these two different systems, we demonstrate the stochastic, nonself-averaged, nature of spin rotation in mesoscopic transport.

**Figure 1:**
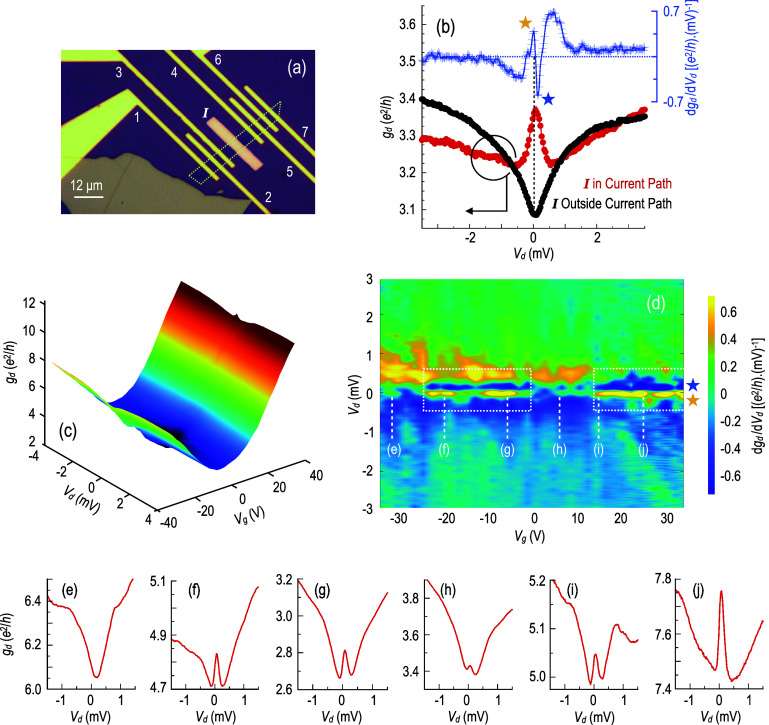
(a) Optical micrograph of the graphene/Co device studied here. Measurement probes are labeled 1–7, I identifies the Co film deposited on top of the graphene. The white dotted line indicates the outline of the mechanically exfoliated monolayer graphene. (b) Left axis: Differential conductance measured at 3 K using different probe combinations. Black filled symbols: current passed between probes 4 and 7. Red filled symbols: current passed between probes 2 and 7. In both cases, voltage is measured between probes 5 and 6. The right axis shows the derivative of the differential conductance curve plotted with red filled symbols. Gate voltage, *V*_g_ = −16 V. (c) Variation of differential conductance with *V*_d_ and *V*_g_. (d) Color contour plotting the variation of the derivative of the differential conductance with *V*_d_ and *V*_g_. White dotted lines enclose ranges of *V*_g_ for which the ZBA is observed. Symbols (★) correspond to the peaks identified in the derivative plot in panel (b). (e–j) Differential conductance at the corresponding gate voltages identified in panel (d). For all of the measurements shown in panels (c–j), current was passed between probes 2 and 7 and voltage was measured between probes 5 and 6 (I in current path).

**Figure 2:**
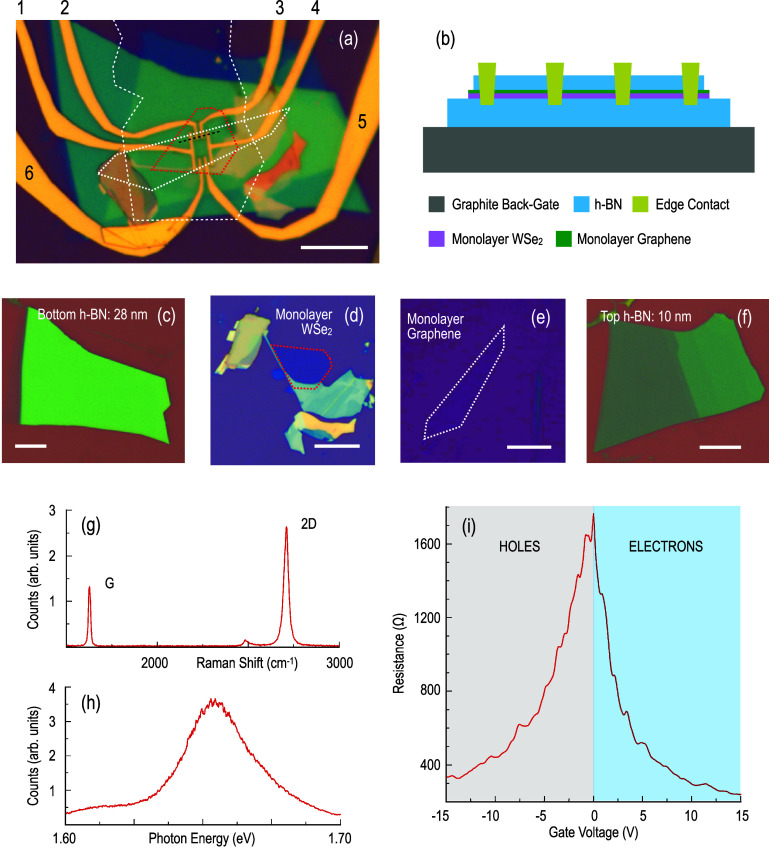
(a) Optical micrograph of the graphene/WSe_2_ device studied here. Measurement probes are labeled 1–6. The white dashed line denotes the pattern of the graphite back gate that the device is fabricated on top of. Red and white dotted lines denote the outline of the monolayer WSe_2_ and graphene, respectively. Spacer bar denotes 10 μm. (b) Schematic cross section of the device, sketched along the direction indicated by the black dashed line in panel (a). (c–f) Optical micrographs of the different components that are stacked to construct the device. Spacer bar denotes 10 μm. (g) Raman spectrum confirms the monolayer nature of the graphene. (h) Photoluminescence confirms the monolayer nature of WSe_2_. (i) The transfer characteristic of the graphene/WSe_2_ heterostructure exhibits an effective resistance modulation at 4 K, with the Dirac point being located close to *V*_g_ = 0 V.

A key feature of the device of Figure 1a is that the Co film (denoted as I) only partially covers the monolayer graphene (the fabrication of these exfoliated devices is described in detail in ref 14). This allows us to compare the measurements of the differential conductance of the same section of graphene (in this case, the section probed by contacts 5 and 6), in configurations in which I lies either outside (current passed between probes 4 and 7) or within (current passed between probes 2 and 7) the current path. The differential conductance itself is measured in a standard approach in which a summing amplifier is used to superimpose a small ac bias (RMS amplitude: 100 μV; frequency: 13 Hz) upon a larger dc component (*V*_d_), enabling the sum of these voltages to be applied to a series combination formed by the device and a standard resistor (for more details, see the Supporting Information of ref 14). Differential conductance [*g*_d_(*V*_d_)] may then be determined from the measurements of the ac voltage drop across the graphene and across the standard (which defines the ac current).

The other system that we study is a graphene/WSe_2_ van der Waals heterostructure, fully encapsulated with hexagonal-boron nitride (h-BN) [Figure 2a–f]. To maximize the influence of the extrinsic SOC, induced in the graphene by the monolayer WSe_2_, the twist angle between the graphene and WSe_2_ layers was set close to 19°, in accordance with the predictions of ref 32 (to configure the system in this manner, the long edge of the graphene crystal [Figure 2e] was used as the reference angle). Electrical contacts to the graphene were realized by means of the one-dimensional edge contact technique, using^33,34^ a combination of electron-beam lithography, reactive-ion etching (in a gas mixture of CHF_3_ and O_2_), and electron-beam evaporation (Cr/Pd/Au: 3/15/80 nm). Prior to assembly of the heterostructure, the monolayer character of both the graphene and WSe_2_ layers was confirmed by Raman spectroscopy [Figure 2g] and photoluminescence^35^ [Figure 2h], respectively (both at room temperature). Our electrical measurements were performed by operating the device in a gating regime in which the WSe_2_ layer should be largely nonconductive, thus ensuring that the conductance of the heterostructure is governed predominantly by that of the graphene layer. Two points can be made here, the first of which relates to the overall resistance of our device. As we show in Figure 2i, the value of this is less than 2 kΩ at all gate voltages, and no more than a few hundred ohms at voltages away from the Dirac point. In contrast, monolayer WSe_2_ should have resistance in the MΩ range (in the absence of sophisticated doping strategies) and thus yield minimal shunting of the graphene. Second, and perhaps more significantly, we point to the pronounced impact of the back-gate voltage on the device. As can be seen in Figure 2i, the transfer curve of the graphene/WSe_2_ system exhibits efficient, symmetric, ambipolar character that would not be expected if there was a significant population of free carriers, capable of mediating gate screening, in WSe_2_.

### 2.2 Graphene/Co

In Figure 1b, we compare the results of two different measurements of the differential conductance of the graphene/Co system. In the first of these, *g*_d_ is determined by passing current between probes 4 and 7, in which configuration the Co film (I) lies outside of the current path. Under such conditions, the differential conductance (black filled symbols) shows a monotonic increase with increasing (positive or negative) *V*_d_, behavior that we have previously demonstrated to be a general feature of the low-temperature differential conductance of graphene.^36^ In that work, the increase of conductance with increasing bias (of either polarity) was shown to be consistent with a suppression of weak localization, in an effect in which the bias essentially mimics the impact of increasing temperature.

In the second measurement (red filled symbols) shown in Figure 1b, differential conductance is determined by passing current between probes 2 and 7, a configuration that now includes I in the current path. In this situation, a clear ZBA is observed in *g*_d_, a feature that is suggestive of anti-localization, rather than weak localization. In Figure 1c, we plot the variation of *g*_d_(*V*_d_, *V*_g_) on both the electron and hole sides of the Dirac point and identify the presence of a fine substructure around *V*_d_ = 0. To highlight this structure more clearly, we compute the derivative of *g*_d_(*V*_d_) and plot this quantity with blue crosses in Figure 1b. Taking this derivative has the effect of removing the large background-conductance variation on either side of *V*_d_ = 0, converting the ZBA into a doublet structure with a sharp maximum and minimum (identified by the yellow and blue stars in the upper part of the figure). In the color contour of Figure 1d, we plot the variation of this derivative for the same range of *V*_d_ and *V*_g_ shown in Figure 1c, configuring the color scale such that the presence of the doublet structure, identified already in Figure 1b, is denoted by intense yellow and blue coloring on either side of *V*_d_ = 0. The areas enclosed by white dotted lines in this contour thus correspond to those ranges of gate voltage for which the doublet structure (and so the ZBA) is present. Outside of these regions, where no ZBA is observed, the differential conductance instead exhibits a local minimum at *V*_d_ = 0. This corresponds to a situation for which d*g*_d_/d*V*_d_ is close to zero near zero bias, a condition denoted by the green coloring in the contour of Figure 1d. Noting these characteristics, the results of Figure 1d reflect a strongly mesoscopic variation of the ZBA as the gate voltage is varied, with the anomaly appearing and disappearing in stochastic fashion as *V*_g_ is changed. Panels (e–j) also highlight this evolution, showing examples of the differential conductance at a few selected gate voltages (as denoted in Figure 1d). It seems clear from these results how the differential conductance undergoes stochastic changes as the gate voltage is varied, with the ZBA fluctuating strongly in amplitude.

While the results of Figure 1 are consistent with those reported previously for another similar device, that earlier experiment^14^ was performed over a relatively narrow range of hole doping. In this work here, in contrast, we show that the ZBA is manifested on both the electron and hole sides of the Dirac point. We also confirm the mesoscopic character of the anomaly, which emerges and disappears in almost random fashion as the gate voltage is varied. The ZBA itself corresponds to an enhancement of the conductance at zero bias and as such points to the role of WAL.^17,18^ This presumably arises from extrinsically induced SOC, generated in the graphene in the region where it is in direct contact with Co (hence the dependence on whether I is included in the measurement circuit or not). Further support for this idea is provided by our observations for the graphene/WSe_2_ system, as we now discuss.

### 2.3 Graphene/WSe_2_

As we illustrate in Figure 2i, the transfer characteristic of the graphene/WSe_2_ heterostructure exhibits an effective resistance modulation at low temperatures, with the Dirac point being located close to *V*_g_ = 0 V (all of the transport measurements that we report for graphene/WSe_2_ were performed while passing current between probes 1 and 4 in Figure 2a and measuring voltage between probes 2 and 3). It is worth pointing out that in this device, WSe_2_ is present underneath the entirety of the graphene layer (see Figure 2a). At the same time, while Co is a ferromagnet that may induce both extrinsic SOC and spin polarization in graphene,^14^ WSe_2_ may serve as a source of SOC^24^ but is a diamagnet (in bulk) with no spin polarization. These differences should therefore provide a valuable contrast with the graphene/Co system discussed above. Another point to be emphasized is that the maximum resistance of around 1.7 kΩ in Figure 2i is much less than *h*/4*e* ^2^, indicating that we are likely not in the quantum spin-Hall state in our system.^37^

In Figure 3a, in analogy with the results of Figure 1d, we plot the differential-conductance contour for the graphene/WSe_2_ device at 0.3 K. In Figure 3b, we show a selected line-plot to illustrate that the differential conductance can exhibit a ZBA, with features reminiscent of those observed for the graphene/Co system. The contour plot of Figure 3a demonstrates that the anomaly exhibits a similar mesoscopic character to that noted earlier, with the peak again emerging and disappearing stochastically as the gate voltage is varied; as before, with our observations for the graphene/Co system, the differential conductance is characterized by regions of gate voltage for which the ZBA is present, separated from ranges for which it is absent. This notion is highlighted by the results presented in the panels of Figure 3c–h.

**Figure 3:**
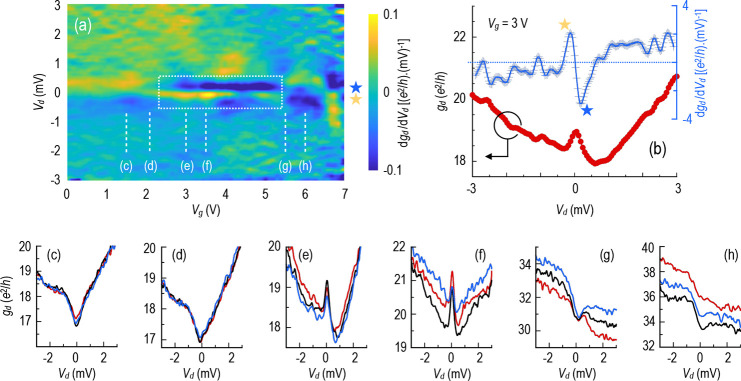
(a) Color contour plotting the variation of the derivative of the differential conductance of graphene/WSe_2_ with *V*_d_ and *V*_g_. Temperature is 0.3 K. White dotted lines enclose ranges for which the ZBA is observed. Symbols (★) correspond to the peaks identified in the derivative plot in panel (b). Red filled symbols (left axis) denote the differential conductance of graphene/WSe_2_ for a gate voltage of 3.0 V. Blue crosses (right axis) show the derivative of the differential conductance. (c–h) Differential conductance at the corresponding gate voltages identified in panel (a). Each such panel shows three such measurements, each separated in gate voltage by 100 mV.

As noted earlier, the ZBA corresponds to a low-temperature enhancement of the zero-bias conductance and, as such, is suggestive of a WAL effect. A key characteristic of WAL is that it is known to be suppressed by a magnetic field.^18^ We have therefore explored the magnetoresistance of the graphene/WSe_2_ system, for a wide range of magnetic fields (−9 T ≤ *B* ≤ + 9 T) applied perpendicular to the plane of 2D transport. At fields in excess of ±1.5 T, we observe clear evidence of Landau-level quantization, leading to Shubnikov-de Haas oscillations in the longitudinal resistance [*R*_*xx*_, see Figure 4a,b] and to quantum plateaus in the Hall conductance [*G*_*xy*_, see Figure 4a,c]; the relatively modest fields at which these effects emerge point to the high quality of this device.

**Figure 4:**
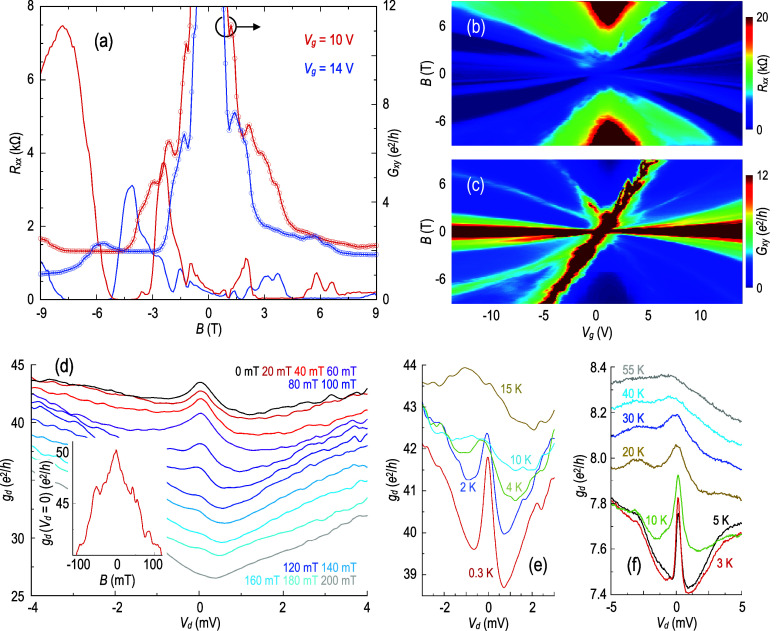
(a) Variation of longitudinal resistance (*R*_*xx*_) and Hall conductance (*G*_*xy*_) with a magnetic field, measured for graphene/WSe_2_ at 0.3 K. (b), (c) Color contours plotting corresponding variation of *R*_*xx*_ and *G*_*xy*_ as a function of *V*_g_ and *B*. (d) Differential conductance plotted at 0.3 K and at various out-of-plane magnetic fields. Inset: Negative magneto-conductance, indicative of antilocalization, measured at 0.3 K. (e) Temperature-dependent evolution of the ZBA in graphene/WSe_2_. (f) Temperature-dependent evolution of the ZBA in graphene/Co.

In Figure 4d, we show the impact of modest magnetic fields on the ZBA. This feature is damped by fields as low as 100 mT (transforming, eventually, into a local minimum in the zero-bias conductance), behavior that is clearly consistent with WAL. Offering further support for this idea, in the inset to the same figure, we plot the results of a measurement of the (low-magnetic-field) magneto-conductance of the graphene/WSe_2_ device; the negative variation of the magneto-conductance exhibited in this plot is a well-known experimental signature of WAL. In Figure 4e, we show the influence of temperature on the ZBA. The peak quickly broadens with an increase of this parameter and is eventually unobservable beyond 10 K. This behavior is reminiscent of that exhibited by the graphene/Co system,^14^ as we indicate in Figure 4f. This shows that, at a gate voltage for which the ZBA is initially prominent (at 3 K), it eventually washes out in the range of 30–40 K, similar to our earlier report for another graphene/Co device.^14^ These observations are also consistent with other experimental reports, which have shown^38–41^ the persistence of weak-localization effects in graphene to such temperatures. It therefore seems that the ZBA in this system is more robust with respect to the increase of temperature than that exhibited by graphene/WSe_2_. We speculate that this reflects the different nature of the induced SOC in these systems: in graphene/Co, the ZBA is measured in native graphene, away from the region (I) where extrinsic SOC is induced; in graphene/WSe_2_, the high-SOC underlayer is in contact with the graphene over the entire region where transport occurs [cf. Figures 1a and 2a]. In spite of these differences, the temperature-dependent decay of the ZBA in both systems is consistent with the influence of increased spin decoherence at higher temperatures.

## 3 Conclusions

In this work, we have addressed the possibility of inducing spin rotation in graphene by placing it in contact with ferromagnetic Co and with diamagnetic WSe_2_. The similar phenomenology exhibited in these two different experiments suggests the possibility of realizing this rotation using very different substrates. One effect associated with placing a 2D material like graphene on any substrate is the breaking of inversion symmetry, due to the presence of the interface between these two materials (i.e., a Rashba-like mechanism). Our measurements of the graphene/Co system demonstrate that this effect alone may not be sufficiently strong to generate the WAL manifested in our experiments. We point here to the behavior in Figure 1b (see the black data points) and to the results of our earlier work,^14^ which show that restricting the current path to a region for which the graphene is in contact with SiO_2_ alone does not generate a ZBA. In this situation, while the SiO_2_ breaks inversion symmetry, the effect of this does not seem to be sufficiently strong, to generate the spin rotation we obtain when either Co or WSe_2_ is in contact with graphene. From this perspective, it would therefore appear that substrates possessing significant SOC (such as WSe_2_), or both SOC and strong spin polarization (Co), are required to induce spin rotation in graphene.

For both systems that we study here, the ZBA exhibits a stochastic variation when the Fermi level is swept through the graphene bands, with ranges for which this feature is either prominent or strongly suppressed. These variations are observed on both the electron and hole sides of the Dirac point [see Figure 1d] and their random character points to a mesoscopic effect. As noted in ref 17, WAL in thin metallic films is a consequence of spin rotation that is generated in materials with strong SOC. In measurements performed on macroscopic scales, the resistance of such films is determined by contributions from electrons that follow a large number of different backscattered trajectories, with a broad distribution of path lengths. Resultant antilocalization arises from the quantum interference of electron partial waves that propagate around these closed loops in opposite directions; when the spin interference is averaged over all such trajectories, the outcome is self-averaging that yields an overall relative phase between the counterpropagating spins that is close to 2π. Since spin-1/2 particles have a rotational periodicity of 4π,^19^ this antilocalization thus reduces the probability of backscattering and leads to an enhancement of the conductance (at zero magnetic field).^18^ Traditionally, such behavior has been demonstrated in magneto-resistance studies, in which the application of the magnetic field breaks time-reversal symmetry and thus suppresses the spin interference (giving rise to a positive magneto-resistance at small fields).

In contrast to the situation described above, our experiments are performed in the mesoscopic limit, where transport is expected to exhibit a coherent character, and the notion of ensemble averaging is no longer necessarily valid. The stochastic character of the ZBA, exhibited when graphene is coupled to both Co and WSe_2_, indeed speaks to a nonself-averaging nature to spin rotation in this limit. Specifically, our experiments suggest that the absence, or incomplete character, of self-averaging can allow spin rotation to be modulated between constructive and destructive, resulting, respectively, in the presence or absence of a ZBA. By demonstrating how spin rotation in mesoscopic systems with strong SOC may be externally gated, our work therefore provides an understanding that may ultimately enable the efficient modulation of spin currents in future spintronic devices.^1,2^

In summary, in this work, we have explored the manifestations of spin rotation in graphene, in proximity with two different types of high-SOC materials (Co and WSe_2_). Using WAL as a probe of the induced rotation, we have demonstrated that spin interference exhibits a highly stochastic (nonself-averaging) character in the mesoscopic limit. At low temperatures (<20 K), the spin rotation is manifested as a zero-bias peak (or ZBA) in the differential conductance, a feature that, as expected for WAL, is suppressed by fairly modest magnetic fields (<∼10^2^ mT). The ZBA moreover exhibits a stochastic variation when a gate voltage is used to sweep the Fermi level through the graphene bands, with ranges for which the antilocalization is either prominent or strongly suppressed. This mesoscopic character is exhibited by both studied systems, whose ZBA is also damped in similar fashion with increasing temperature. Our demonstrations provide fundamental insight into the nonensemble-averaged character of spin interference in mesoscopic systems with strong SOC and, more specifically, into how the details of spin rotation are impacted by external gating. This understanding may ultimately enable the efficient modulation of spin currents in future spintronic devices.^1,2^

## 4 Experimental Methods

### 4.1 Graphene/Co Device Fabrication

Graphene/Co devices were fabricated by exfoliating Kish graphite onto a doped Si substrate with a 300 nm SiO_2_ cap layer and an underlying, heavily doped, Si layer that served as a back gate. A two-step electron-beam lithography (EBL) process (with subsequent lift-off steps) was used to define Cr/Au (5/75 nm) measurement electrodes and the 20 nm thick Co element. Graphene layer identification was achieved through a combination of optical microscopy and Raman imaging. Thermal annealing was undertaken at two different stages during the fabrication process, with the first step being performed in a H_2_/Ar atmosphere (at 400 °C) prior to EBL of the Cr/Au measurement probes. Following this, and before the EBL step used to define the magnetic element, rapid thermal annealing was performed at 250 °C for 10 min under vacuum. Electron-beam evaporation of Co was used to deposit the magnet on the graphene and was performed after pumping the deposition chamber for 4 h to reach a pressure of ∼10^–7^ mbar. Finally, the devices were wire-bonded, mounted in our cryostat, and annealed at 100 °C for 12 h while maintaining a vacuum of 5 × 10^–5^ Torr.

### 4.2 Graphene/WSe_2_ Device Fabrication

The graphene/WSe_2_ van der Waals heterostructure was fabricated with full encapsulation with h-BN [Figure 2a–f]. The procedure that we used to realize this structure involved the use of a PC/PDMS (where PC is 6% poly bisphenol A carbonate dissolved in chloroform and PDMS is polydimethylsiloxane) stamp to perform dry transfer.^42^ In Figure 2c–f, we show the different layers that were used to assemble the heterostructure shown schematically in Figure 2b. The bottom h-BN layer [Figure 2c] was first transferred onto a graphite back-gate [dashed line in Figure 2a], following which the remaining layers were added by stacking (as we have described previously^33,34,43,44^ ). Electrical contact to the graphene layer was made by using reactive-ion etching to expose the edges of the graphene sheet. This process is known^45^ to oxidize the exposed WSe_2_ (while leaving the graphene unaffected), forming an amorphous WO_*x*_ edge layer that, in turn, is expected to prevent the formation of an ohmic contact to the monolayer WSe_2_.

### 4.3 Electrical Measurements

The electrical measurements reported in this study were performed in a four-terminal geometry, allowing us to eliminate the influence of contact/lead resistance. For the measurements of the differential conductance (*g*_d_), a DC voltage (*V*_d_) of varying amplitudes was added on top of a small AC component (typically ∼100 μV at a frequency of around 13 Hz), allowing us to measure *g*_d_ as a function of both *V*_d_ and gate voltage. We refer the reader to our previous work^14^ for further details of this setup.

## Data Availability

The data that support the findings of this study are available from the corresponding authors upon reasonable request.
